# Changes in Walking Stability at Different Percentages of Preferred Walking Speed in Healthy Young and Older Adults: Insights From Movement Component Analysis

**DOI:** 10.1155/tswj/9971520

**Published:** 2025-02-12

**Authors:** Arunee Promsri

**Affiliations:** Department of Physical Therapy, School of Allied Health Sciences, University of Phayao, Phayao 56000, Thailand

**Keywords:** age-related change, comfortable walking speed, gait, movement synergy, neuromuscular control

## Abstract

Walking instability increases the risk of falls and compromises mobility safety. This study aimed to explore the impact of various percentages of preferred walking speed (PWS)—specifically, 40%, 55%, 70%, 85%, 100%, 115%, 130%, and 145%—along with age-related changes, on walking stability during treadmill walking. Kinematic marker data from all walking speed trials were pooled for analysis, involving a total of 26 participants (13 young adults aged 24.7 ± 2.4 years and 13 older adults aged 60.8 ± 6.4 years). These pooled data were then decomposed into various movement components (i.e., movement strategies), known as principal movements (PMs), using principal component analysis (PCA). These PMs, which resemble the phases of a gait cycle, collectively contribute to the accomplishment of the walking task. The participant-specific largest Lyapunov exponent (LyE) was employed to assess the local dynamic stability of individual PMs, with lower LyE values indicating higher stability, thereby allowing for the examination of walking speed and age effects. The main findings revealed that only the effects of altered walking speeds were observed; specifically, the LyE value for the midstance phase (PM_3_) at 100% of PWS was significantly lower than at 40% of PWS (*p*=0.001), and there was a trend indicating that the LyE value at 100% of PWS was also lower than at 140% of PWS (*p*=0.027). These results suggest that PWS enhances the stability of the mid-stance-phase movement component of the gait cycle more than the slower and faster walking speeds during treadmill walking.

## 1. Introduction

Walking is a fundamental part of daily activities, and a participant's preferred walking speed (PWS) is commonly used in gait analysis to reflect typical walking patterns [1]. Also referred to as “spontaneous” or “self-selected” walking speed, PWS represents the natural pace individuals choose during everyday activities and is often seen as an appropriate exercise intensity for weight reduction programs [2]. It serves as a well-recognized measure of overall gait performance and is frequently utilized to assess locomotor capability [3]. Unlike a fixed speed, PWS is preferred in the study of human walking because each individual has their own unique pace [4], which tends to slow down with age [5]. Defining walking speed as a percentage of PWS offers several benefits, such as enabling comparisons across different speeds while allowing individuals to maintain control. This approach effectively standardizes gait parameters, accounting for age and physical factors [6].

When considering walking stability, the ability to walk without falling despite perturbation can be evaluated using various methods [7]. Nonlinear methods, e.g., local dynamic stability measured by calculating the largest Lyapunov exponent (LyE), have been employed to assess the ability to adapt to minor internal or external disturbances, ensuring effective locomotion [8]. Stability, defined as the capacity of a system (e.g., the movement system) to maintain its original state despite internal (e.g., neuromuscular) and external (e.g., environmental) disruptions [7], provides insights into the variability in motor task performance and measures the effectiveness of dynamic error correction [9]. Regarding local dynamic stability, it refers to the neuromuscular system's ability to manage tiny perturbations during locomotion [10]. In walking, reduced local dynamic stability in weight-bearing movement components among healthy older adults has been linked to an increased risk of falls [11]. Additionally, diminished running stability has been associated with a decreased ability to compensate for minor perturbations, potentially leading to overuse injuries, e.g., bone stress injuries caused by repetitive and monotonous loading exceeding the capacity of the bones [12, 13].

Since effective completion of any motor task requires cooperative contributions from whole-body segments [14], analyzing whole-body movements during locomotion can enhance our understanding of how walking tasks are adaptively performed. Research has shown that motor activities involve various movement synergies that work together to achieve specific task goals [14]. These task-dependent movement synergies can adjust to both internal and external demands [15]. Dimensionality reduction techniques, e.g., principal component analysis (PCA), have become popular for decomposing movement synergies from whole-body movements [14, 16]. PCA addresses redundancy issues in motor function by reducing the number of features necessary to complete a task, generating fewer new variables, and retaining most of the original information about how people move [14]. Specifically, PCA applied to kinematic data produces a set of one-dimensional movement components or synergies known as “principal movements” (PMs) [17]. Information about the position and acceleration of individual PMs reflects their direct connection to forces in the system and myoelectric activity, providing an effective assessment of neuromuscular control of individual movement strategies [18, 19]. In this context, applying LyE to the positions of individual PMs can help quantify the stability of the collective movement strategies employed during walking tasks [3]. These measures also indicate the ability to handle minor disturbances (e.g., minor slips or trips) detected from a consistent walking pattern without any external disruptions apart from those inherent to the testing environment or the system itself [7].

Although using a percentage of PWS to define walking speed effectively standardizes gait parameters for age and physical factors [6], the influence of walking at various percentages of PWS on gait stability, particularly among young and older healthy adults, remains uncertain. Investigating how changes in walking speed affect gait stability is crucial for fully understanding human movement and guiding interventions to improve walking performance in various situations. The ability to adjust speed during walking is an important mechanism that requires varying levels of muscular activity to adapt appropriately to changes in task demands [3]. Walking instability has been correlated with fall risk in the healthy elderly [11], and older adults typically display greater instability than young adults [20]. Lower physical performance caused by the inherent degenerative decline in muscle strength has been involved in this instability [11]. Moreover, the inherent slower walking speed in elderly subjects may be associated with a cautious gait strategy [21], typically marked by mild to moderate slowing, reduced stride length, and mild widening of the base of support [22]. It is intriguing to explore whether individuals of different age groups demonstrate similar walking stability when walking at different percentages of PWS.

In summary, the primary objective of the current study was to investigate the influence of altered walking speeds at different percentages of PWS on walking stability. It was hypothesized that PWS would demonstrate greater stability compared to slower [23] and faster [24] walking speeds. Additionally, the study aimed to explore the effects of age-related changes on walking stability. It was anticipated that age-related changes in walking stability would be evident due to inherent alteration in gait parameters, including reduced stride length and frequency, anterior pelvic tilt, decreased plantarflexion, and diminished propulsive mechanical power generation of the trailing limb during the stance phase [5]. These factors are thought to contribute to the decline in PWS [5], with slower walking leading to increased instability [23]. Furthermore, declines in strength and range of motion (ROM) in older adults have also been linked to increased walking instability compared to young adults [25]. It was also expected that the effects of altered walking speed and age would emerge in the specific movement components, e.g., the movement component resembling the single-limb support phase of a gait cycle that has been reported to involve instability found in the healthy older adults and related to increasing the risk of falls [11]. This investigation may provide valuable insights into how the neuromuscular system adapts to different walking speeds and how these adaptations influence gait stability, which is essential for assessing human locomotion and designing interventions to enhance walking stability.

## 2. Materials and Methods

### 2.1. Participants

The kinematic marker data, with no missing markers, were checked and retrieved from the C3D files available in an open-access dataset by Fukuchi et al. [26]. The walking data were collected at the Laboratory of Biomechanics and Motor Control at the Federal University of ABC, Brazil [26]. The study was approved by the university's ethics committee (CAAE: 53063315.7.0000.5594), and all participants provided informed consent prior to participation [26]. Participants met the inclusion criteria, which required them to be within the specified age range of 20–75 years, have no history of lower extremity injury in the six months before data collection and be free of orthopedic or neurologic conditions that could affect gait patterns [26]. Exclusion criteria included recent lower extremity surgeries or injuries, diagnosed orthopedic, neurologic, or cardiovascular conditions that might impair mobility or balance, and the use of medications affecting gait stability [26]. The original dataset provided data for 42 volunteers [26]. However, for the current study, the dataset was narrowed to 13 healthy young adults (6 males and 7 females) with an average age of 24.7 ± 2.4 years and 13 healthy older adults (6 males and 7 females) with an average age of 60.8 ± 6.4 years. Participants with missing markers or incomplete walking trials were excluded. All participants in the final sample completed walking speed trials at 40%, 55%, 70%, 85%, 100%, 115%, 130%, and 145% of their PWS. The sample size for the current study was validated using a priori power analysis with G∗Power, assuming an effect size of 0.25 and a power of 0.95, which indicated that 24 participants would be sufficient.  Table 1 summarizes the participants' characteristics.

The experimental procedures were thoroughly outlined by Fukuchi, Fukuchi, and Duarte [26]. Briefly, a marker-set protocol consisting of 26 anatomical cluster-reflective markers was applied to the pelvic and both lower extremity segments [27]. Prior to data collection, participants familiarized themselves with the treadmill speed by walking at their comfortable pace for 5 min [26]. Subsequently, each participant completed a series of eight gait-speed conditions (40%, 55%, 70%, 85%, 100%, 115%, 130%, and 145% of their self-selected speed, represented by the Froude number), with the trials presented in a randomized order [26]. Participants walked barefoot on a dual-belt, instrumented treadmill (FIT; Bertec, Columbus, OH, USA) at these varying speeds, and the sequence of walking trials was randomized using a computerized random-number generator [26]. After each treadmill task, participants were given adequate time to recover before proceeding to the next walking speed condition. To assess exertion levels, participants' overall perceived exertion was measured using the Borg (6–20) Perceived Exertion Scale [28] to ensure that they did not experience excessive fatigue during the trials [26]. The Borg scale scores after each walking trial did not exceed levels that would prevent participants from continuing with the subsequent walking conditions [26]. Kinematic marker data were collected at a frequency of 150 Hz using a motion capture system (Cortex software version 6.0; Motion Analysis, Santa Rosa, CA, USA), equipped with 12 cameras (Raptor-4; Motion Analysis Corporation, Santa Rosa, CA, USA) [26]. Before the data collection, a standing anatomical calibration trial was conducted to ensure accurate tracking of the markers during the walking trials [26]. During this calibration, participants stood still in a neutral position, and marker trajectories were recorded to establish a reference for the subsequent motion data [26]. The procedure involved aligning the anatomical markers with the participant's body segments to ensure proper tracking, thereby minimizing measurement errors and ensuring that the motion capture system accurately tracked the markers in relation to the participants' movements throughout the gait trials [26].

### 2.2. Movement Synergy Extraction

The data processing for this study was conducted using MATLAB version 2023a (MathWorks Inc., Natick, MA, USA). For each dataset, only 13 markers placed on prominent anatomical landmarks of the lower extremity (anterior superior iliac spine (ASIS), posterior superior iliac spine (PSIS), iliac crest, greater trochanter, head of the fibula, heel, lateral malleolus, and the first metatarsal bone) were selected for further analysis due to their ability to sufficiently frame the shape of the lower extremities [11]. Each dataset produced 39 spatial coordinates (*x*, *y*, and *z*) representing multidimensional posture vectors [14]. To prepare the data, these posture vectors were centered by subtracting the mean posture vector to account for differences in marker positioning and then normalized to the mean Euclidean distance to address anatomical variations. The preprocessed datasets were combined to create a single input matrix (1 trial × 8 walking speed conditions × 26 participants) for subsequent PCA to compare results between walking speed conditions.

PCA was conducted using the PManalyzer software [14], utilizing a singular-value decomposition of the covariance matrix. This process transformed kinematic data into a set of orthogonal eigenvectors known as “principal components” (PC_k_), where the subscript *k* denotes the order of movement components. Each orthogonal eigenvector could generate an animated stick figure referred to as PM_k_ [14], representing its movement pattern closely associated with phases of the gait cycle, for example, the swing and stance phases [19, 29]. Furthermore, the time evolution of each PM was measured by the PC scores, which represented the positions in posture space, or the vector space spanned by the PC eigenvectors. Additionally, principal accelerations (PA_k_) could be calculated from the PC scores using conventional differentiation rules [14]. Previous research has indicated that PA_k_ is correlated with leg myoelectric activity [15], suggesting that PA-based variables can offer insights into the neuromuscular control of individual PM_k_ [29].

To mitigate noise amplification introduced during differentiation, a Fourier analysis was conducted on the raw PC scores [18, 19, 29]. The analysis revealed that the highest power was within the frequency range of 2–4 Hz, with noticeable power still present in the 5–7 Hz range. Therefore, a third-order zero-phase 7-Hz low-pass Butterworth filter was applied to the PCA-based time series before the differentiation step. Additionally, leave-one-out cross-validation was conducted to evaluate the robustness of individual PM_k_ and the PCA-based dependent variables, ensuring the validity of the input data matrix. This study selected the first five PCs that remained robust after cross-validation to test the hypotheses.

### 2.3. PCA-Based Dependent Variables

Two variables based on PCA were computed for each PM. Firstly, the participant-specific relative explained variance (rVAR) of PP_k_ (*t*) or PP_k__rVAR was determined to measure the contribution percentage of each PM to the total variance in postural positions. This calculation provides insights into the significance of each PM_k_ within the overall coordinative movement structures during overground walking [17]. Differences in PP_k__rVAR, for example, those between different walking speeds or age groups, would indicate variations in the coordination structure of the overall postural movements.

Additionally, the participant-specific largest LyE of PP_k_ (*t*) or PP_k__LyE was computed to measure the rate of divergence of nearby trajectories in state space, representing the motor system's ability to mitigate minor perturbations in each PM [18, 19, 29]. A higher LyE value suggests a diminished ability of the motor system to dampen perturbations [10], resulting in increased divergence of state space trajectories and reduced individual walking stability. The LyE calculation involves the application of Wolf's algorithm [30] with specific parameters, including time delay (*τ* = 10) and embedding dimension (*m* = 4) [9]. Embedding dimension and time delay were chosen based on previous studies to optimize the sensitivity of LyE in detecting subtle stability changes during treadmill walking [10]. These parameter values are determined using the average mutual information (AMI) [9] and false nearest neighbor algorithms [31].

### 2.4. Statistical Analysis

Statistical analyses were performed using IBM SPSS Statistics software version 26.0 (SPSS Inc., Chicago, IL, USA), with the significance level set at *α* = 0.05. The Shapiro–Wilk test was used to assess the normality of the data. A repeated-measures ANOVA was applied to examine the effects of walking speeds (within-subject effects) and age (between-subject effects). When significant main effects of walking speeds were identified, Bonferroni post hoc tests were conducted to determine specific differences, with the alpha level adjusted to *α* = 0.006 to account for multiple comparisons. Additionally, Pearson correlation tests were performed to evaluate the relationships between LyE values of PM_1–5_ and walking speeds at various percentages of PWS.

## 3. Results

**Figure 1:**
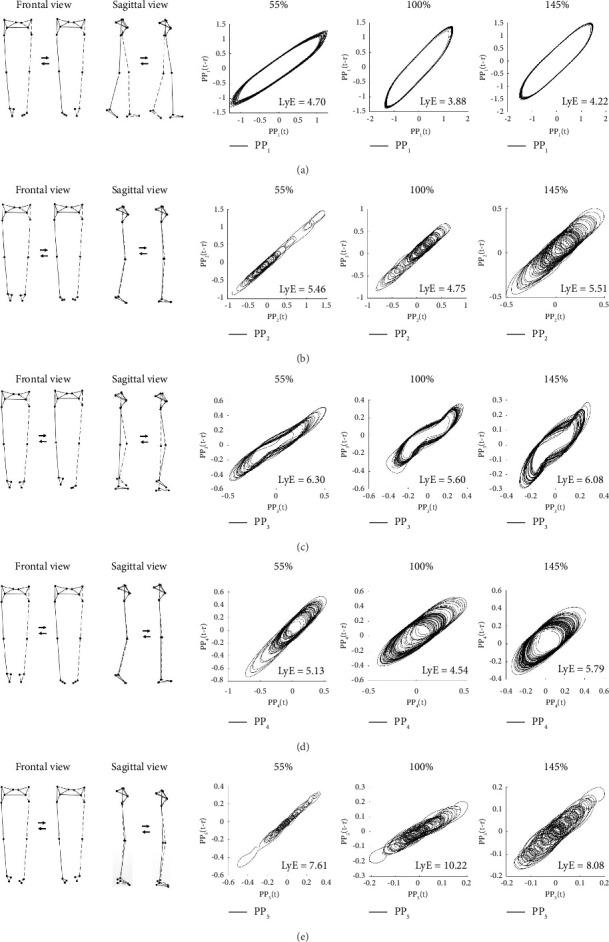
Visualizations of (a) PM_1_, (b) PM_2_, (c) PM_3_, (d) PM_4_, and (e) PM_5_ extracted from the treadmill walking movement and the examples of their corresponding space–time representation for the computed largest Lyapunov exponent (LyE) of individual PP_k_ derived from walking with 55%, 100%, and 155% of preferred walking speed, respectively. Note: LyE data are derived from one female participant. The dashed line indicates the left limb.

Table 2 presents the descriptive characteristics of the first five PMs (PM_1–5_), which collectively account for 99.9% of the total position variance (PP_k__rVAR) and 65.7% of the acceleration variance (PA_k__rVAR). These PM_k_ components correspond to key gait phases: PM_1_ represents the swing phase with antiphase arm and leg movements in the sagittal plane; PM_2_, the double-leg support phase involving weight acceptance and combined knee, vertical body, and treadmill motions; PM_3_, the midstance phase with knee movements and a lateral upper-body shift; PM_4_, vertical ankle and knee flexion/extension; and PM_5_, the single-limb support phase with antiphase hip flexion/extension. These components highlight the coordination required for walking. Visualizations of PM_1–5_ are shown in Figure 1.

### 3.1. Walking Speed Effects

The effects of walking speeds at different percentages of PWS on the compositions (PP_k__rVAR) of individual PMs were only observed in PM_1_, representing the swing-phase movement of the gait cycle (*F*_(2.68,  64.35)_ = 28.28, *p* < 0.001, *η*_*p*_^2^ = 0.541, 1 − *β* = 1); PM_3_, representing the midstance phase of the gait cycle (*F*_(2.17,  58.31)_ = 91.61, *p* < 0.001, *η*_*p*_^2^ = 0.792, 1 − *β* = 1); and PM_4_, representing both ankle and knee flexion and extension (*F*_(2.97,  70.24)_ = 91.89, *p* < 0.001, *η*_*p*_^2^ = 0.793, 1 − *β* = 1).

**Figure 2:**
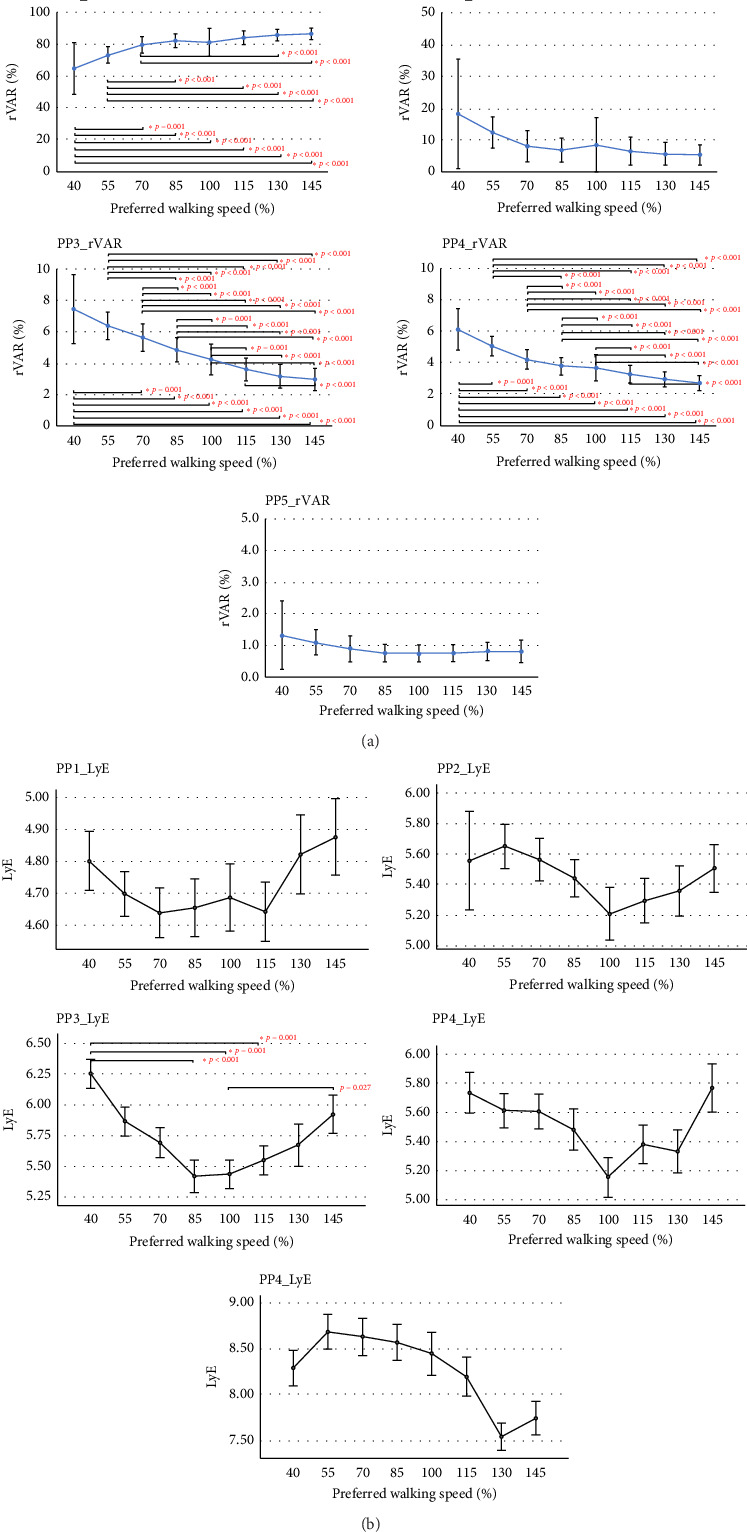
The relative explained variance ((a) PP_k__rVAR) and the largest Lyapunov exponent ((b) PP_k__LyE) of the first five principal movements (PM_1–5_) computed from different percentages of preferred walking speed regarding the walking speed effects (mean ± SD, ⁣^∗^*p* < 0.001).

Figure 2(a) displays the post hoc tests, revealing the significant difference between pairs of walking speeds. For PM_1_ (PP_1__rVAR), the contribution of 40% PWS is lower than walking speeds at 70% (*p* < 0.001), 85% (*p* < 0.001), 100% (*p* < 0.001), 115% (*p* < 0.001), 130% (*p* < 0.001), and 145% (*p* < 0.001) of PWS; walking speed at 55% of PWS is lower than walking speed at 85% (*p* < 0.001), 100% (*p* < 0.001), 115% (*p* < 0.001), 130% (*p* < 0.001), and 145% (*p* < 0.001) of PWS; walking speed at 70% of PWS is lower than walking speed at 130% (*p* < 0.001) and 145% (*p* < 0.001) of PWS.

For PM_3_ (PP_3__rVAR), walking speed at 40% of PWS has greater contribution than walking speeds at 70% (*p* < 0.001), 85% (*p* < 0.001), 100% (*p* < 0.001), 115% (*p* < 0.001), 130% (*p* < 0.001), and 145% (*p* < 0.001) of PWS; walking speed at 55% has greater contribution than walking speeds at 85% (*p* < 0.001), 100% (*p* < 0.001), 115% (*p* < 0.001), 130% (*p* < 0.001), and 145 (*p* < 0.001) of PWS; walking speed at 70% has greater contribution than walking speeds at 85% (*p* < 0.001), 100% (*p* < 0.001), 115% (*p* < 0.001), 130% (*p* < 0.001), and 145 (*p* < 0.001) of PWS; 85% has greater contribution than 100% (*p* < 0.001), 115% (*p* < 0.001), 130% (*p* < 0.001), and 145 (*p* < 0.001) of PWS; walking speed at 100% of PWS has greater than walking speeds at 115% (*p* < 0.001), 130% (*p* < 0.001), and 145 (*p* < 0.001) of PWS; and walking speed at 115% of PWS has greater than walking speeds at 145% of PWS.

For PM_4_ (PP_4__rVAR), walking speed at 40% of PWS has greater than walking speeds at 70% (*p* < 0.001), 85% (*p* < 0.001), 100% (*p* < 0.001), 115% (*p* < 0.001), 130% (*p* < 0.001), and 145 (*p* < 0.001) of PWS; walking speed at 55% of PWS is greater than walking speeds at 85% (*p* < 0.001), 100% (*p* < 0.001), 115% (*p* < 0.001), 130% (*p* < 0.001), and 145 (*p* < 0.001) of PWS; walking speed at 70% of PWS is greater than 85% (*p* < 0.001), 100% (*p* < 0.001), 115% (*p* < 0.001), 130% (*p* < 0.001), and 145 (*p* < 0.001) of PWS; walking speed at 85% of PWS is greater than walking speeds at 100% (*p* < 0.001), 115% (*p* < 0.001), 130% (*p* < 0.001), and 145 (*p* < 0.001) od PWS; walking speed of 100% of PWS is greater than walking speeds at 115% (*p* < 0.001), 130% (*p* < 0.001), and 145 (*p* < 0.001) of PWS; and walking speed at 115% of PWS is greater than walking speeds at 145% of PWS.

In addition, the effects of walking speeds at different percentages of PWS on local dynamic stability (PP_k__LyE) of individual PMs were only observed in PM_3_, representing the midstance phase of the gait cycle (*F*_(1.97,  47.29)_ = 11.50, *p* < 0.001, *η*_*p*_^2^ = 0.324, 1 − *β* = 1).  Figure 2(b) displays the post hoc tests, revealing a significant difference between pairs of walking speeds. Walking speed at 40% of PWS showed less stability (higher LyE) compared to walking speeds at 85% (*p* < 0.001), 100% (*p*=0.001), and 115% (*p*=0.001). Additionally, walking at 100% of PWS exhibited a trend of higher stability compared to faster walking speeds at 145% of PWS.

### 3.2. Age Effects

No age effect is observed in the compositions of individual PMs (PP_1__rVAR (PM_1_) *F*_(1, 24)_ = 0.034, *p*=0.856, *η*_*p*_^2^ = 0.001, 1 − *β* = 0.054; PP_2__rVAR (PM_2_) *F*_(1, 24)_ = 0.064, *p*=0.803, *η*_*p*_^2^ = 0.003, 1 − *β* = 0.057; PP_3__rVAR (PM_3_) *F*_(1, 24)_ = 0.602, *p*=0.445, *η*_*p*_^2^ = 0.024, 1 − *β* = 0.116; PP_4__rVAR (PM_4_) *F*_(1, 24)_ = 1.842, *p*=0.187, *η*_*p*_^2^ = 0.071, 1 − *β* = 0.256; and PP_5__rVAR (PM_5_) *F*_(1, 24)_ = 1.468, *p*=0.160, *η*_*p*_^2^ = 0.081, 1 − *β* = 0.285). Additionally, no age effects appeared in local dynamic stability of individual PMs (PP_1__LyE (PM_1_) *F*_(1, 24)_ = 0.327, *p*=0.573, *η*_*p*_^2^ = 0.013, 1 − *β* = 0.085; PP_2__LyE (PM_2_) *F*_(1, 24)_ = 0.108, *p*=0.745, *η*_*p*_^2^ = 0.004, 1 − *β* = 0.061; PP_3__LyE (PM_3_) *F*_(1, 24)_ = 0.089, *p*=0.768, *η*_*p*_^2^ = 0.004, 1 − *β* = 0.059; PP_4__LyE (PM_4_) *F*_(1, 24)_ = 0.985, *p*=0.331, *η*_*p*_^2^ = 0.039, 1 − *β* = 0.159; and PP_5__LyE (PM_5_) *F*_(1, 24)_ = 1.477, *p*=0.236, *η*_*p*_^2^ = 0.058, 1 − *β* = 0.215).

Moreover, no interaction effects between walking speeds and age are observed in the composition of individual PMs (PP_1__rVAR (PM_1_) *F*_(7, 168)_ = 1.620, *p*=0.133, *η*_*p*_^2^ = 0.063, 1 − *β* = 0.659; PP_2__rVAR (PM_2_) *F*_(7, 168)_ = 1.423, *p*=0.195, *η*_*p*_^2^ = 0.056, 1 − *β* = 0.594); PP_3__rVAR (PM_3_) *F*_(7, 168)_ = 0.250, *p*=0.972, *η*_*p*_^2^ = 0.010, 1 − *β* = 0.122; PP_4__rVAR (PM_4_) *F*_(7, 168)_ = 0.059, *p*=0.992, *η*_*p*_^2^ = 0.007, 1 − *β* = 0.094; and PP_5__rVAR (PM_5_) *F*_(7, 168)_ = 1.645, *p*=0.126, *η*_*p*_^2^ = 0.064, 1 − *β* = 0.667). In addition, no interaction effects between walking speeds and age emerge in the local dynamic stability of individual PMs (PP_1__rVAR (PM_1_) *F*_(7, 168)_ = 0276, *p*=0.962, *η*_*p*_^2^ = 0.011, 1 − *β* = 1.934; PP_2__rVAR (PM_2_) *F*_(7, 168)_ = 1.355, *p*=0.228, *η*_*p*_^2^ = 0.053, 1 − *β* = 0.565; PP_3__rVAR (PM_3_) *F*_(7, 168)_ = 1.115, *p*=0.356, *η*_*p*_^2^ = 0.044, 1 − *β* = 0.471; PP_4__rVAR (PM_4_) *F*_(7, 168)_ = 1.452, *p*=0.188, *η*_*p*_^2^ = 0.057, 1 − *β* = 0.601; and PP_5__rVAR (PM_5_) *F*_(7, 168)_ = 1.356, *p*=0.227, *η*_*p*_^2^ = 0.053, 1 − *β* = 0.566).

### 3.3. Correlations Between Local Dynamic Stability and Walking Speed

The correlations between LyE values and walking speeds at different percentages of PWSs are summarized in Table 3. At 40% PWS, no significant correlations are observed. At 55% PWS, significant negative correlations are identified for LyE_PP_2_ (*r* = −0.404, *p*=0.041, moderate) and LyE_PP_4_ (*r* = −0.464, *p*=0.017, moderate). At 70% PWS, significant negative correlations are noted for LyE_PP_3_ (*r* = −0.391, *p*=0.049, moderate) and LyE_PP_5_ (*r* = −0.538, *p*=0.005, strong). At 85% PWS, LyE_PP_3_ shows a significant negative correlation (*r* = −0.502, *p*=0.009, strong), which is also observed at 100% PWS (*r* = −0.476, *p*=0.014, moderate). At 115% PWS, a significant negative correlation is found for LyE_PP_5_ (*r* = −0.484, *p*=0.012, moderate). No significant correlations are observed at 130% PWS, but at 145% PWS, LyE_PP_5_ shows a significant negative correlation (*r* = −0.467, *p*=0.016, moderate). Overall, LyE_PP_3_ and LyE_PP_5_ consistently exhibit significant negative correlations at various walking speeds, particularly within the 55%–115% PWS range, with correlations ranging from moderate to strong, highlighting their sensitivity to changes in walking speed in this range.

## 4. Discussion

The present study investigated the effects of altered walking speeds and age-related changes on walking stability assessed by applying the largest LyE to individual treadmill walking movement components (PMs), which were decomposed using PCA in healthy young and older adults. The results indicate that only the effects of altered walking speeds by different percentages of PWS were observed in specific PM_3_, resembling the mid-stance-phase movement of the gait cycle.

Regarding the effects of altered walking speed, increased walking speeds modulated the gradually increased contribution of the swing-phase movement (PM_1_) and decreased contribution of the stance-phase movements (PM_3–4_) of the gait cycle. However, changes in stability were observed only in the mid-stance-phase component (PM_3_). The LyE values for this movement component at 100% of PWS exhibited a significant reduction compared to slower speeds (i.e., 40% of PWS). Interestingly, a trend toward lower LyE values during faster walking speeds, reaching up to 145% of the PWS, was also observed. Additionally, a V-shaped graph is observed for the LyE, with 100% and 85% of PWS at the base of the V-shaped graph, highlighting that PWSs enhance more stability than slower and faster walking. The current findings were in line with previous reports that lower [23] and faster [24] walking speeds decreased stability during walking. Focusing on the midstance phase is pivotal to maintaining stability and forward movement, which requires more metabolic cost than the swing and double support phases of the gait cycle [32]. This finding may link to an increased risk of falls, since PM_3_ resembles the midstance phase that is associated with the lateral weight shift and could increase instability in the mediolateral direction, which is a predictor of falls [33, 34]. Moreover, complex neuromuscular adjustments occur within lower limb muscles and joints as the body's center of mass shifts over the standing leg [35]. Variations in LyE values of PM_3_ during the midstance phase reveal the intricate response to changes in walking speed, highlighting the complex relationship among gait stability, mechanical forces, and neural control mechanisms.

Unexpectedly, the current study found no significant difference in PWS between healthy young and older adults, consistent with Kang and Dingwell [20]. This contrasts with the findings of Chung and Wang [6] and Winter et al. [21], which reported that PWS decreases with increasing age. The absence of age-related differences in walking stability, despite similar walking speeds across age groups, as also seen in a previous study [20], suggests that factors other than speed may influence stability. One possible factor is the age of older adults in the current study, which is relatively young compared to a previous study that reported older adults exhibited higher instability of the trunk roll across all walking speeds (80%–120% of PWS) than young adults, indicating greater instability that persisted even after adjusting for declines in strength and ROM [25]. This may be because the average age of the previous participants (72 ± 6 years) [25] was older than that of the current participants (60 ± 6 years). Findings from earlier studies showing increased instability in older adults, especially in the mediolateral direction measured from trunk acceleration, begin from ages 40–50 [36], may not fully apply to our sample. Another critical factor to consider is the body mass index (BMI) of participants. In our study, older adults had significantly higher body mass compared to younger adults. A previous study reported that an increase in BMI could lead to a decline in dynamic stability and dynamic balance ability, suggesting that obesity adversely affects dynamic stability, increasing fall risk [37]. Therefore, while both age groups demonstrated similar walking speeds, the higher BMI in older adults may have influenced their dynamic stability differently, potentially offsetting the expected age-related decline in stability. Additionally, variations in measurement methods and body parts for assessing dynamic stability may contribute to the differences observed across studies (e.g., the trunk acceleration in Kang and Dingwell [20] and Terrier and Reynard [36] and lower limb kinematics in the current study). However, for future research, further exploring how BMI influences movement components and local dynamic stability of individual movement components of walking, particularly in older populations with varying age ranges, is of interest.

Interestingly, correlations between local dynamic stability (LyE) and walking speeds at different percentages of PWS were observed in specific movement components (PMs). Significant negative correlations emerged between 55% and 115% of PWS, particularly for LyE_PP3, which corresponds to the midstance phase of the gait cycle. This suggests that as walking speed increases within the 55%–115% range of PWS, local dynamic stability improves (lower LyE values), indicating a more controlled and predictable walking pattern [10, 19, 29]. The decrease in LyE suggests that the body maintains a more stable and coordinated gait as walking speed increases, likely due to more efficient biomechanical control [10]. Furthermore, walking speeds within the 55%–115% PWS range (0.6–1.4 m/s) may be particularly beneficial in promoting stability, especially for the elderly, individuals recovering from injury, or those with balance impairments. Maintaining walking speeds within this range could help older adults or individuals at risk of falls sustain stability and control during the single-leg support phase of walking [29].

From a clinical perspective, encouraging individuals to walk at their natural pace may optimize gait stability during weight-bearing phases, thereby reducing fall risk, particularly since instability during the midstance phase of the gait cycle is associated with an increased risk of falls [11]. Training should focus on muscle groups involved in the midstance phase, such as hip extensors, knee flexors, and frontal-plane muscles like hip abductors, which contribute to mediolateral stability [38]. These muscles are crucial for leg movement during terminal swing (i.e., deceleration of the leg in preparation for heel contact) and stabilization following heel contact during initial stance [35]. The observed trend of reduced stability at both slower and faster walking speeds highlights the importance of maintaining an optimal gait speed. By optimizing walking speed and strengthening key muscles involved in the stance phase, practitioners can significantly enhance gait stability, reducing fall risks and improving overall quality of life, especially for elderly adults.

### 4.1. Limitations and Future Research

The current study, a secondary data analysis conducted in a controlled experimental setting, is limited in its applicability to real-world situations and broader populations. Future research should validate these findings across diverse groups and environmental conditions. Differences between treadmill and overground walking biomechanics also warrant attention, as prior studies report consistent temporal gait parameters but significant variations in joint mechanics and muscle activation patterns, such as reduced dorsiflexor and knee extensor moments and increased hip extensor moments during treadmill walking [39].

A further limitation is the exclusive use of the LyE to assess local dynamic stability, without analyzing time-related kinematic measures like joint angles and angular velocities at the hip, knee, and ankle. Incorporating these metrics could enhance understanding of joint contributions to walking stability across speeds.

This study did not observe age-related effects on local dynamic stability, in contrast to prior research reporting significant differences in gait parameters, such as the gait cycle, peak joint angle timing, and angular velocity, between elderly and very elderly women [40]. A potential explanation for this discrepancy could be the younger age of the older adults in the current study. However, focusing on movement components or synergies that incorporate how all body segments work together to achieve the walking task may provide deeper insights into gait dynamics [29]. Future research should explore a broader age range, integrate stability metrics with detailed kinematic analyses, and include diverse populations and real-world walking conditions to better understand these dynamics and improve the generalizability of findings.

## 5. Conclusion

The current study investigated local dynamic stability (assessed by LyE) during treadmill walking at various percentages of PWS in healthy young and older adults. The results showed that individuals walking at their preferred speed exhibited greater stability during the midstance phase (PM_3_) compared to those walking either too slowly or too quickly. Moreover, the negative correlations between LyE values of PM_3_ (reflecting mediolateral sway during the midstance phase) and walking speeds within the 70%–100% PWS range (approximately 0.85–1.25 m/s) suggest that walking at these speeds enhances single-leg support stability. These findings highlight the importance of individualized walking speeds for effective fall prevention and gait rehabilitation, ultimately improving mobility and quality of life.

## Figures and Tables

**Table 1:** Characteristics of participants (mean ± SD, ⁣^∗^*p* < 0.05, and ⁣^∗∗^*p* < 0.001).

	Young adults	Older adults	*p* value
Age (years)	24.7 ± 2.4	60.8 ± 6.4	< 0.001⁣^∗∗^
Mass (kg)	64.9 ± 9.9	66.3 ± 11.8	0.004
Height (m)	171.7 ± 8.6	160.8 ± 9.0	0.737
Body mass index (kg/m^2^)	22.0 ± 3.3	25.5 ± 3.6	0.016⁣^∗^
Walking speeds (m/s)			
40% of PWS	0.50 ± 0.04	0.49 ± 0.07	0.839
55% of PWS	0.69 ± 0.06	0.67 ± 0.11	0.622
70% of PWS	0.87 ± 0.07	0.86 ± 0.12	0.742
85% of PWS	1.06 ± 0.08	1.05 ± 0.15	0.756
100% of PWS	1.25 ± 0.10	1.23 ± 0.18	0.773
115% of PWS	1.44 ± 0.11	1.42 ± 0.20	0.751
130% of PWS	1.62 ± 0.13	1.60 ± 0.23	0.762
145% of PWS	1.81 ± 0.15	1.78 ± 0.26	0.751

**Table 2:** The descriptive characteristics of the first five principal components (PM_1–5_) and their eigenvalue (%, mean ± SD) derived from pooling all walking speed trials together.

PM_*k*_	Descriptive characteristics	PP_k__rVAR
*k* = 1	The swing phase: antiphase arm and leg movements in the sagittal plane	79.73 ± 10.52
2	The double-leg support movement: weight acceptance during double-leg support involving a combination of knee flexion/extension movements and vertical whole-body movements, coupled with the treadmill's sliding motion.	9.07 ± 9.02
3	The single-limb support (midstance) phase: antiphase knee flexion and extension movements combined with a lateral shift of the upper body onto the stance leg	4.79 ± 1.85
4	Both ankle and knee flexion and extension movements in the vertical direction	3.96 ± 1.31
5	The single-limb support phase: antiphase hip flexion and extension movements	0.89 ± 0.56

*Note: k* indicates the order of movement components.

**Table 3:** Correlations between local dynamic stability (LyE) and walking speeds at various percentages of preferred walking speed (PWS).

		LyE_PP_1_	LyE_PP_2_	LyE_PP_3_	LyE_PP_4_	LyE_PP_5_
*40% of PWS*						
Walking speed	*r*	0.285	−0.046	−0.275	−0.210	0.004
	*p* value	0.158	0.824	0.174	0.303	0.983

*55% of PWS*						
Walking speed	*r*	0.439	−0.404	0.033	−0.464	−0.102
	*p* value	0.025⁣^∗^	0.041⁣^∗^	0.873	0.017⁣^∗^	0.619

*70% of PWS*						
Walking speed	*r*	−0.026	−0.014	−0.391	0.041	−0.538
	*p* value	0.901	0.948	0.049⁣^∗^	0.843	0.005⁣^∗^

*85% of PWS*						
Walking speed	*r*	−0.091	−0.299	−0.502	−0.272	−0.290
	*p* value	0.660	0.139	0.009⁣^∗^	0.178	0.150

*100% of PWS*						
Walking speed	*r*	−0.202	−0.084	−0.476	−0.187	−0.297
	*p* value	0.323	0.682	0.014⁣^∗^	0.359	0.141

*115% of PWS*						
Walking speed	*r*	−0.293	0.246	−0.384	−0.088	−0.484
	*p* value	0.146	0.226	0.053	0.669	0.012⁣^∗^

*130% of PWS*						
Walking speed	*r*	0.039	0.315	0.019	0.065	−0.330
	*p* value	0.851	0.117	0.925	0.752	0.099

*145% of PWS*						
Walking speed	*r*	0.196	0.347	0.142	0.155	−0.467
	*p* value	0.338	0.082	0.489	0.449	0.016⁣^∗^

⁣^∗^*p* < 0.05 (2-tailed).

## Data Availability

The data analyzed in this study are available at http://10.6084/m9.figshare.5722711.v2 (accessed on February 12, 2022) by Fukuchi, Fukuchi, and Duarte [26].
